# Longitudinal association between binge eating and metabolic syndrome in adults: findings from the ELSA-Brasil cohort

**DOI:** 10.1111/acps.13356

**Published:** 2021-08-16

**Authors:** Francesca Solmi, Arlinda B. Moreno, Glyn Lewis, Maria Angélica Nunes, Maria de Jesus Mendes da Fonseca, Rosane Harter Griep

**Affiliations:** 1 Division of Psychiatry, University College London, London, UK; 2 Department of Epidemiology and Quantitative Methods in Health, National School of Public Health, Oswaldo Cruz Foundation, Rio de Janeiro, Brazil; 3 Postgraduate Program in Epidemiology, School of Medicine, Federal University of Rio Grande do Sul, Porto Alegre, RS, Brazil; 4 Laboratory of Health and Environment Education, Oswaldo Cruz Institute, Oswaldo Cruz Foundation, Rio de Janeiro, Brazil

**Keywords:** binge eating, metabolic syndrome, cohort study, ELSA-Brasil

## Abstract

**Objective:**

Individuals with bulimia nervosa and binge eating disorder have greater cardiovascular morbidity than the general population. Longitudinal research on the association between binge eating and metabolic syndrome is limited. We tested the longitudinal association between binge eating and metabolic syndrome and its components in a large population sample of Brazilian adults.

**Methods:**

We used data from Brazilian Longitudinal Study of Adult Health (ELSA-Brasil, N=15,105). To test for the association between binge eating at baseline (2008-2010) and metabolic syndrome at follow-up (2012-2014), we used univariable and multivariable logistic regression models progressively adjusting for potential socio-demographic confounders, and number of metabolic syndrome components, and body mass index (BMI) at baseline.

**Results:**

In total, 13,388 participants (54.8% female; 52.2% white) had complete data on all variables of interest. Binge eating was associated with increased odds of metabolic syndrome at follow-up (odds ratio (OR):1.66, 95% confidence intervals (CI): 1.44,1.75). However, the size of this association was attenuated after including number of metabolic syndrome components at baseline (OR:1.19, 95%CI: 1.05,1.35), and was no longer present after adjusting for baseline BMI (OR:1.09, 95%CI: 0.96, 1.02). Binge eating was also associated with higher odds of hypertension (OR:1.14, 95%CI: 0.99,1.37) and hypertriglyceridemia (OR:1.21, 95%CI: 1.06,1.37) at the follow-up assessment after adjustment for all confounders.

**Conclusions:**

Individuals who binge eat are at increased risk of metabolic syndrome via increased BMI, and of hypertriglyceridemia and hypertension independently of BMI. If these are causal associations, effective interventions for binge eating could also have beneficial effects on metabolic health outcomes.

## Introduction

The metabolic syndrome is a condition characterised by increased central adiposity, hypertension, hypertriglyceridemia, elevated fasting blood glucose, and low levels of serum high-density lipoprotein (HDL) cholesterol. On average, individuals with metabolic syndrome have a two-fold increase in the risk of cardiovascular disease and mortality.^1^ Identifying modifiable risk factors is therefore key to successful preventative measures and interventions to reduce cardiovascular risk in the population.

Recent evidence points to increased cardiovascular morbidity in people with binge eating disorder^2^ and bulimia nervosa.^3^ Both of these eating disorder diagnoses are characterised by the presence of recurrent episodes of binge eating, defined as instances when a person eats large amounts of food in a short period of time while experiencing a sense of loss of control. Although longitudinal studies investigating the association between binge eating, and metabolic syndrome and its components are now needed to begin to understand pathways to cardiovascular risk in these populations, only a handful of such studies exist.^4–7^

To date, the literature suggests that binge eating ^4,5^, binge eating disorder^7^, or disordered eating behaviours (including binge eating)^6^ are longitudinally associated with greater odds of metabolic syndrome, with this association largely ^6,7^ or entirely^4,5^ explained by higher average body mass index (BMI) in these populations. As adolescent samples find that binge eating is associated with an increased risk of having a BMI in the overweight or obese range in adulthood^8,9^, it may be possible that addressing binge eating behaviours could help to prevent a proportion of metabolic syndrome cases by avoiding excessive weight gain. However, two small studies have found BMI-independent longitudinal associations between binge eating and increased odds of dyslipidemia/ hypertriglyceridemia, in both children and adults,^4,7^ and larger study of adults an association with higher fasting glucose.^5^ This suggests that correct identification and treatment of binge eating could yield long-term positive physical health outcomes regardless of a person’s weight.

However, not only is research into these associations scant, but all of the existing studies also have important limitations which limit inferences that can be made from their results. All but one^5^ had small sample sizes (range n=115 to n=268) ^4,6,7^ which can reduce statistical power to detect associations. Some studies only included adults who had an overweight or obese BMI^6^ or adolescents at high risk for adult obesity^4^, potentially resulting in selection bias. These studies also did not account for use of medications in their definition of metabolic syndrome, so they might have underestimated the association. Finally, all of these studies relied on predominantly Caucasians samples (i.e. >95%) based in Europe or North America, so that little is known of these associations in other ethnic groups and regions.^4–7^

### Aims of the study

The aim of this study was therefore to investigate the longitudinal association between binge eating and both metabolic syndrome and its individual components in a large prospective study of Brazilian adults.

## Methods

### Sample

We used data from the Brazilian Longitudinal Study of Adult Health (ELSA-Brasil). ELSA-Brasil is an ongoing multicenter cohort study which recruited 15,105 (95.5% of those invited to participate, n=15,821) civil servants aged from 34 to 75 years from research and teaching public institutions in six of Brazil’s state capitals (Belo Horizonte, Porto Alegre, Rio de Janeiro, Salvador, São Paulo, and Vitória) between 2008 and 2010. The cohort’s aim is to investigate the incidence and progression of diabetes mellitus and cardiovascular diseases, as well as to examine the biological, behavioral, environmental, occupational, psychological, and social factors associated with these diseases and their complications in a Brazilian context. The ELSA-Brasil design, sampling procedures, construction of the questionnaire, quality assurance, and control measures have been described in detail previously.^10,11^

In this study we included participants who had complete data on the exposure of interest, as well as confounder and outcome data, the latter collected at phase two of ELSA-Brasil (2012-2014). As a sensitivity analysis, we also ran our models in a sample of participants with complete exposure and imputed confounders and outcome.

### Outcome

The primary outcome of these analyses was presence of metabolic syndrome at follow-up. In line with harmonised consensus criteria guidelines,^12^ we defined metabolic syndrome as having at least three of the following components: (i) high waist circumference (>=90 cm in men and >=80 cm in women); (ii) high blood glucose (>=100 mg/dl or use of oral hypoglycemic drugs or insulin); (iii) low HDL cholesterol (<40 mg/dl for men and <50 mg/dl for women or drugs for reduced HDL-C); (iv) hypertriglyceridemia (TAG >=150 mg/dl or use of drugs to treat elevated triglycerides); (v) hypertension (blood pressure >=130/85mmHg or antihypertensive treatment). As secondary outcomes, we also used a variable indicating number of metabolic syndrome components at follow-up (range: 0 to 5) and each metabolic syndrome component individually.

### Exposure

At baseline assessment participants were asked the following question: “Some people, at certain times, eat a large amount of food at once, in a short time (up to two hours). They feel they have lost control over eating, that is, they cannot avoid starting to eat, and after starting, cannot stop. During the past 6 months, how often did you eat this way?” Possible answers were: never; less than once a week; once a week; or twice a week. In line with DSM-5^13^ diagnostic criteria for bulimia nervosa and binge eating disorder, we considered binge eating present when participants reported episodes of binge eating occurring at least once per week.

### Confounders

In eFigure 1, we show our causal assumptions using a Direct Acyclic Graph (DAG) which we used to identify potential confounders to adjust our analyses for so as to estimate the total effect of binge eating at baseline on metabolic syndrome at follow-up. We adjusted our models for a number of socio-demographic variables which were self-reported by participants at baseline assessment. These were: sex (male or female); a continuous indicator of age; race/skin color based on Brazil’s population census classification (Black, Brown (“Pardo”), White, Asian or Indigenous); marital status (married, living with a partner, previous married, single, and widowed at baseline); occupational class (based on occupation, classified as Manual routine, Manual non-routine, Non-manual routine, Non-manual non-routine at baseline); smoking (never, in the past, currently), drinking alcohol (never, in the past, currently). We also included an indicator of common mental disorders as a potential confounder. Common mental disorders were measured at baseline using the total score of the Clinical Interview Schedule Revised (CIS-R), which was administered by trained interviewers.^14,15^ This scale ranges from zero to 57 with higher scores indicating greater symptoms. The CIS-R was culturally adapted to be used in the Brazilian population and translated into Portuguese.^15^

As can be seen in our DAG (eFigure 1), in light of previous evidence longitudinally linking binge eating to increases in BMI^8,9^ and metabolic syndrome ^4–7^, we hypothesized that these two might be on the causal pathway between our exposure and outcome (eFigure 1a). However, an alternative hypothesis could be that participants with higher BMI or metabolic syndrome could have been advised to diet and this could have triggered binge eating, or that higher BMI could result in increased appetite, as suggested by a recent mendelian randomization study^16^ (eFigure 1b). Since these were measured at the same time of the exposure, and we could not tease out the temporality of these associations, we further adjusted our models for BMI (weight in kilograms/height in meters^2^) and outcome measurement (e.g.: number of metabolic syndrome components) at baseline in separate models.

### Statistical analysis

We first described the analytical sample with respect to the exposure and confounders using frequencies with proportions and means with standard deviations. We tested for the association between binge eating at baseline and metabolic syndrome at follow-up using a univariable and three multivariable logistic regression models progressively adjusting for: all socio-demographic variables and CIS-R score at baseline (model 1); number of metabolic syndrome components at baseline (model 2); and baseline BMI (model 3). In model 3, we further included an interaction term between binge eating and sex to test for the presence of sex differences in the association between binge eating and metabolic outcomes. We subsequently stratified analyses by sex if there was evidence of an interaction. As secondary analyses, we ran the same logistic regression models using each of the metabolic syndrome components as outcomes. When investigating these outcomes, in model 2 we adjusted for the corresponding symptom at baseline as opposed to number of metabolic syndrome components.

We also investigated the association between binge eating at baseline and number of metabolic syndrome components at follow-up as outcome using negative binomial regression models (as there was over-dispersion in the outcome variable) progressively building our models as in the main analyses. Finally, to explore whether differences in follow up time between participants could have biased our results, we also additionally adjusted all our models for time to follow up.

We ran all our main analyses on participants with complete data on all variables included in the model. As sensitivity analyses, we imputed missing outcome and confounder data using multiple imputation by chained equations imputing 50 datasets. In our imputation models we used all variables included in our models as well as a number of auxiliary variables, as recommended.^17^ We ran all of our analyses in Stata15.^18^

### Ethics approval

ELSA-Brasil is a multicenter study; therefore, the project was approved by the Research Ethics National Committee (Comitê Nacional de Ética em Pesquisa) and by the committees of each institution involved in December 2008 (Study registration number = 140/08). The volunteers gave written consent to participate.

## Results

### Sample & missing data

Of the 15,105 participants who were initially recruited into the ELSA-Brasil cohort, 15,074 (99.8%) had complete exposure data. Of these, 246 (1.6%) died prior to the second wave of data collection and 992 (6.5%) were lost to follow up, leaving 13,836 participants of whom 13,388 (96.7%) had data available on all variables of interest (flowchart of participation in eFigure 2). Mean follow up time in the sample was 3.85 years (standard deviation [SD]: 0.42) and this did not differ between exposed (mean: 3.85, SD: 0.41) and unexposed (mean: 3.85, SD: 0.44, p=0.26) participants. Participants with metabolic syndrome at follow up had slightly longer follow up time (mean: 3.86, SD: 0.43) compared to those without metabolic syndrome (mean: 3.85, SD: 0.41, p=0.12, data not presented in tables).

As shown in Table 1, the majority of the participants: were women (54.8%) and of white ethnicity (52.2%); had a university degree (53.0%); and were from a non-manual/non-routine social class (53.5%); were current drinkers (69.7%), and had never smoked (57.8%). At baseline, 26.8% of the sample had metabolic syndrome.

Men, older participants, those with metabolic syndrome, lower education and those who were current or past smokers were more likely to be lost to follow up or to have died. Participants with higher BMI and higher CIS-R scores were more likely to have been lost to follow up. (eTable 1)

### Frequency of binge eating

A total of 2,048 (15.3%) participants reported experiencing episodes of binge eating occurring at least weekly in the previous six months at baseline. As shown in Table 1, binge eating was more common among women; participants of black ethnicity; those who were divorced, separated, or widowed; and among past smokers or drinkers. Participants who reported binge eating were younger, had a greater BMI and CIS-R scores, and had a higher prevalence of metabolic syndrome at baseline.

### Binge eating and the metabolic syndrome

The prevalence of metabolic syndrome at follow up was greater among those who experienced weekly episodes of binge eating (40.9%) compared to those who did not (30.3%, Table 2). In the univariable model, participants with binge eating had greater odds of having metabolic syndrome at follow up (odds ratio (OR) 1.59, 95% confidence intervals (CI): 1.44 to 1.75). This association persisted in model 1 adjusting for socio-demographic covariates and CIS-R score (OR: 1.66, 95%CI: 1.50 to 1.84). The magnitude of this association was attenuated by the inclusion of number of metabolic syndrome components at baseline in model 2 (OR: 1.19, 95%CI: 1.05 to 1.35), and after controlling for BMI at baseline in model 3 (OR: 1.09, 95%CI: 0.96 to 1.25) where there was no longer evidence of an association. There was no evidence of an interaction between sex and binge eating. We observed similar patterns when using number of components at follow-up as outcomes. There was evidence that participants with binge eating had more metabolic syndrome components at follow-up in the univariable model (coefficient: 0.19, 95%CI: 0.16 to 0.22) and in models adjusting for socio-demographic variables (coefficient: 0.20, 95%CI: 0.16 to 0.22) and number of metabolic syndrome components at baseline (coefficient: 0.06, 95%CI: 0.03 to 0.09), although here the magnitude of the association was reduced. After further adjusting for BMI at baseline there was no longer evidence of an association (coefficient: 0.02, 95%CI: -0.01 to 0.06, Table 2). Inclusion of time to follow up in our models did not affect results either when using a binary indicator of metabolic syndrome (OR: 1.09, 95%CI: 0.96 to 1.25) or number of metabolic syndrome components as outcomes (coefficient: 0.02, 95%CI: -0.01 to 0.06, result not presented in table).

### Binge eating and individual components of the Metabolic syndrome

As shown in Table 3, in univariable models, binge eating was associated with greater odds of each of the outcomes and these associations were still present after adjusting for socio-demographic variables and CIS-R scores in model 1 adjusting for socio-demographic characteristics and model 2 adjusting for baseline values of the outcome. After further adjustment for baseline BMI in model 3, there was evidence of an association between binge eating and hypertriglyceridemia (OR: 1.21, 95%CI: 1.07 to 1.37) and hypertension (OR:1.14, 95%CI: 0.99 to 1.31). Results did not vary when including follow up time in the models for hypertriglyceridemia (OR: 1.21, 95% CI: 1.07 to 1.37) and blood pressure (OR: 1.14, 95%CI: 0.99 to 1.32, results not presented in table). There was evidence of an interaction between sex and binge eating in the association between binge eating and hypertriglyceridemia (p=0.020). Men who reported binge eating had greater odds of hypertriglyceridemia (OR: 1.38, 95%CI: 1.15 to 1.68) compared to those who did not, but not women (OR: 1.09, 95%CI: 0.92 to 1.29).

### Sensitivity analyses

When we re-ran all of our analyses in a sample of participants with complete exposure and imputed confounders and outcome (n=14,828) results did not change (eTable 2 and 3). However, as the sample of these analyses was larger – thus conferring greater statistical power – we observed slightly stronger evidence of an association between binge eating and greater odds of hypertension (OR: 1.15, 95%CI: 1.00 to 1.32, p=0.055).

## Discussion

### Main findings

In this large population study of Brazilian civil servants, we found that those who reported weekly episodes of binge eating had greater odds of having metabolic syndrome at follow up after accounting for baseline socio-demographic variables, common mental disorders, and number of metabolic syndrome components. However, it appears that this association was explained by the fact that those with binge eating had a higher BMI on average. We also found that, across the five metabolic syndrome components, participants who reported binge eating had greater odds of hypertriglyceridemia and hypertension at follow up, even after accounting for BMI and presence of these outcomes at baseline. Although, for hypertension evidence of an association was weaker compared to that observed for hypertriglyceridemia it is possible that could have been due to low statistical power, as increased sample size in sensitivity analyses resulted in a stronger association. Finally, we found some evidence of a differential association between binge eating and hypertriglyceridemia between men and women which warrants further investigation.

This study adds to the existing literature by showing that previously reported associations between binge eating and components of the metabolic syndrome are not only observed in clinical populations, populations with higher BMI, and those of white ethnicity. On the contrary, these associations are also observed in a population sample of civil servants across a range of BMI values and ethnic groups.

### Interpretation of findings and implications.

Our findings are in line with those of other studies showing that the effect of binge eating in increasing the odds of metabolic syndrome is largely explained by differences in BMI.^4–6^ Existing evidence has shown that binge eating leads to weight gain over time.^8,19^ Hence, we believe that it is plausible that BMI is one mechanism linking binge eating and increased risk of metabolic syndrome in this population.^8,19^ Our results, however, suggests this might not be the only pathway to cardiovascular risk. We observed that binge eating was associated with higher odds of hypertriglyceridemia and, to a lower extent, hypertension – established risk factors for cardiovascular disease^20^ – at all levels of BMI. The association between binge eating and hypertriglyceridemia has also been previously reported in several, but not all^5^, samples of children and adults^4,7^ and is biologically plausible.

There is evidence that food consumed during binge eating episodes in both bulimia nervosa and binge eating disorder is typically high in carbohydrates and fats (with more carbohydrates consumed on average).^21–23^ Carbohydrate-induced hypertriglyceridemia has been previously described, with studies showing a dose-response association between carbohydrates consumption and elevations in plasma triglycerides.^24,25^ General population studies find that dietary carbohydrates in children’s and adults’ diet are associated with elevated blood pressure and dyslipidemia.^26–28^ It has also been hypothesized that triglycerides might induce leptin-resistance. ^29,30^ This could suggest that binge eating-induced metabolic changes could also exacerbate weight gain, resulting in greater risk of developing metabolic syndrome via increased BMI over time, as we observed in our results.

We also observed that the association between binge eating and hypertriglyceridemia was greater in male compared to female participants. Other longitudinal studies did not investigate this question, so comparisons are difficult to make. However, one cross-sectional study of treatment-seeking adults with binge eating disorder observed similar associations. ^31^ The authors suggested that differences in treatment-seeking behaviours could account for these differences. Another possibility is that women might be more commonly reporting subjective binge eating^5,32^ and that this might explain the weaker association between binge eating and hypertriglyceridemia.

### Strengths and limitations

This study has a number of strengths. As far as we are aware, this is the largest study to date investigating the prospective association between binge eating and metabolic syndrome in an ethnically diverse sample. We were able to control for a large number of socio-demographic, socio-economic, behavioral, biological, and psychological variables therefore minimizing the potential for residual confounding. Attrition in this sample was minimal; the majority of participants had complete information on all confounders and was retained in the study at follow-up. When we imputed missing data, our results were entirely consistent with those based on complete cases thus supporting the robustness of our results. Although our sample was constituted by civil servants employed in six higher education and research institutions, previous studies have shown that the sample is largely representative of the Brazilian population.^33^ Finally, we had data available not only on serum levels of biomarkers relevant to the diagnosis of metabolic syndrome, but we also had information on whether participants were taking any medications affecting those values, which we could use in our definition of the outcome.

Nevertheless, some limitations ought to be mentioned. Our measure of binge eating could not distinguish between objective and subjective episodes of binge eating, and might thus have resulted in some measurement error. Subjective binge eating refers to episodes when an individual might perceive a sense of loss of control over eating, but they do not consume an objectively large amount of food. This could have resulted in an under-estimation of the association with the outcomes of interest. The question used to measure binge eating was not taken from a specific eating disorder questionnaire. Nevertheless, it covers core diagnostic criteria used in DSM-5 namely overeating and loss of control over the amount of food eating, it is similar to questions employed in other studies ^34,35^, and has been previously used in other studies using this cohort ^36,37^. We used binary indicators of metabolic syndrome and its constituent components to define our outcome measures and this could have resulted in loss of statistical power and information, as well as in type I error^38^. Although using continuous outcome indicators would have addressed these potential limitations, our approach allowed us to include in our outcome definition participants who were taking medications affecting the values of the metabolic syndrome components under study, thus avoiding underestimating associations. Time between baseline and follow up assessments ranged from two to six years, and, although including time to follow up in our models did not affect results, we cannot fully exclude that right censoring due to differences in follow up length could have occurred. We adjusted for whether participants never smoked or drank alcohol, did so in the past, or at baseline because these variables allowed us to control for lifetime behaviors, i.e. including those prior to baseline exposure measurements. This categorization did not allow us to have a more granular understanding of the effect that controlling for the amount of current smoking and drinking might have on the association between binge eating and metabolic syndrome. Nevertheless, we observed that BMI and number of metabolic syndrome components at baseline were the strongest confounders of the association under study, for which we did not find overall strong evidence for. Hence, it is unlikely that number of cigarettes or amount of alcohol drank would have altered these findings, particularly as this would have meant collapsing the ‘never’ and ‘past’ categories which might have different risk profiles.

We adjusted our models for BMI and metabolic syndrome at baseline, both of which – based on our causal assumptions – might lie on the causal pathway between binge eating and metabolic syndrome. For this reason, we controlled for BMI and baseline metabolic syndrome in two separate models so that model 1 would reflect the total effect of binge eating on metabolic syndrome at follow up (if our causal assumptions are correct) and model 2 and 3 would reflect the total effect of binge eating under the competing assumption that high BMI and metabolic syndrome lead to binge eating. Future studies should aim at disentangling these associations. An important question that remains to be answered is around the mediating role of changes in BMI over time in this association. As our second measurement of BMI was concurrent to outcome measurement, we could not have disentangled temporal associations hence we did not attempt any mediation models. Future studies with more than two waves of data collection available should investigate these associations.

## Conclusions

Binge eating is difficult to diagnose, as people might not disclose these behaviors because of feelings of guilt or shame. Because people who experience binge eating can have higher BMI, they are often referred to weight loss programs, which might in fact exacerbate binge eating symptoms, as food restriction is a trigger for loss of control over eating.^39^ It is therefore crucial that primary care physicians are trained in recognising and diagnosing binge eating across the whole BMI spectrum, so that people who experience these symptoms can be referred to effective psychological treatments^40^. If the associations that we have observed are causal, reducing binge eating could have the beneficial effect of preventing cardiovascular outcomes in the long term at all levels of BMI.

## Supplementary Material

Supplemental material

## Figures and Tables

**Table 1:** Sample characteristics, participants with complete exposure data (n=13,388)

	Total n(%)	Binge eating at least once a week		
		No N(%)	Yes N(%)	p-value
	13,388	11,340 (84.7%)	2,048 (15.3%)	
**Sex**				
*Male*	6,047 (45.2%)	5,279 (87.3%)	768 (12.7%)	
*Female*	7,341 (54.8%)	6,061 (82.6%)	1,280 (17.4%)	<0.0001
**Ethnicity**				
*Black*	2,159 (16.1%)	1,778 (82.3%)	381 (17.7%)	
*Pardo*	3,754 (28.1%)	3.165 (84.3%)	589 (15.7%)	
*White*	6,992 (52.2%)	5,981 (85.5%)	1,011 (14.5%)	
*Asian or indigenous*	483 (3.6%)	416 (86.1%)	67 (13.9%)	0.003
**Highest education**				
*No schooling*	722 (5.4%)	601 (83.2%)	121 (16.8%)	
*Elementary school*	878 (6.6%)	741 (84.4%)	137 (15.6%)	
*Secondary school*	4,694 (35.0%)	3,870 (82.4%)	824 (17.6%)	
*University degree*	7,094 (53.0%)	6,128 (86.4%)	966 (13.6%)	<0.0001
**Marital status**				
Married	576,602 (49.3%)	5,678 (86.0%)	924 (14.0%)	
Partner	2,296 (17.2%)	1,944 (84.7%)	352 (15.3%)	
Separated/divorced	2,591 (19.4%)	2,145 (82.8%)	446 (17.2%)	
Single	1,367 (10.2%)	1,139 (83.3%)	228 (16.7%)	
Widowed	532 (3.9%)	434 (81.6%)	98 (18.4%)	<0.0001
**Social Class**				
Manual-routine	2,171 (16.2%)	1,811 (83.4%)	360 (16.6%)	
Manual non-routine	169 (1.3%)	148 (87.4%)	21 (12.6%)	
Non-manual routine	3,885 (29.0%)	3,199 (82.3%)	686 (17.7%)	
Non-manual non-routine	7,163 (53.5%)	6,182 (86.3%)	981 (13.7%)	<0.0001
**Smoker**				
Never smoker	7,740 (57.8%)	6,599 (82.3%)	1,141 (14.7%)	
Past smoker	3,966 (29.6%)	3,317 (83.6%)	649 (16.4%)	
Current smoker	1,680 (12.6%)	1,424 (84.7%)	258 (15.3%)	0.07
**Alcohol use**				
Never drank	1,412 (10.6%)	1,176 (83.3%)	236 (16.7%)	
Past drinker	2,640 (19.7%)	2,158 (81.7%)	482 (18.3%)	
Current drinker	9,336 (69.7%)	8,006 (85.8%)	1,330 (14.3%)	<0.0001
**Metabolic syndrome baseline**				
*No*	9,794 (73.2%)	8,450 (86.3%)	1,344 (13.7%)	
*Yes*	3,594 (26.8%)	2,890 (80.4%)	704 (19.6%)	<0.0001
**Hypertension baseline**				
*No*	8,487 (63.4%)	7,235 (85.3%)	1,252 (14.7%)	
*Yes*	4,901 (36.6%)	4,105 (83.7%)	796 (16.3%)	0.02
**Hypertriglyceridemia baseline**				
No	9,820 (73.4%)	8,429 (85.8%)	1,391 (14.2%)	
Yes	3,568 (26.7%)	2,911 (81.6%)	675 (18.4%)	<0.0001
**High fasting glucose baseline**				
No	11,305 (84.4%)	9,655 (85.4%)	1,650 (14.6%)	
Yes	2,083 (15.6%)	1,685 (80.9%)	398 (19.1%)	<0.0001
**Low HDL cholesterol baseline**				
No	9,873 (73.7%)	8,493 (86.0%)	1,380 (14.0%)	
Yes	3,515 (26.3%)	2,847 (81.0%)	668 (19.0%)	<0.0001
**High waste circumference baseline**				
No	4,143 (31.0%)	3,834 (92.5%)	309 (7.5%)	
Yes	9,245 (69.0%)	7,506 (81.2%)	1,739 (18.8%)	<0.0001
	**Mean (SD)**	**Mean (SD)**	**Mean (SD)**	
**Number of metabolic syndrome constituent components at baseline**	1.7 (1.3)	1.7 (1.3)	2.1 (1.2)	<0.0001
**Age**	51.8 (9.0)	52.0 (9.0)	50.3 (9.4)	<0.0001
**Body Mass Index**	26.9 (4.7)	26.6 (4.5)	29.3 (4.9)	<0.0001
**CIS-R-total score**	8.2 (8.0)	7.6 (7.5)	11.7 (9.4)	<0.0001

List of abbreviations: CIS-R = Clinical Interview Schedule – Revised, SD = standard deviation

**Table 2:** Prevalence of Metabolic syndrome at ELSA-Brasil follow-up among those who did and did not experience weekly episodes of binge eating at baseline. Univariable and multivariable logistic regression models of the association between binge eating at baseline and Metabolic syndrome at follow-up. Sample based on participants with complete data (n=13,388)

	Outcome: Metabolic syndrome at follow up		
	**n (%)**		**Odds ratio (95%CI)**
	**Binge eating absent**	**Binge eating present**	
Crude model	3,437 (30.32%)	837 (40.87%)	1.59 (1.44 to 1.75), p<0.0001
Adjusted model 1	-		1.66 (1.50 to 1.84), p<0.0001
Adjusted model 2	-		1.19 (1.05 to 1.35), p=0.008
Adjusted model 3	-		1.09 (0.96 to 1.25), p=0.191
Binge eating*sex interaction p-value			0.754
	**Mean (standard deviation)**		**Coefficient^a^ (95%CI)**
	**Binge eating absent**	**Binge eating present**	
Crude model	1.89 (1.28)	2.29 (1.24)	0.19 (0.16 to 0.22), p<0.0001
Adjusted model 1			0.20 (0.16 to 0.23), p<0.0001
Adjusted model 2			0.06 (0.03 to 0.09), p<0.0001
Adjusted model 3			0.02 (-0.01 to 0.06), p=0.167
Binge eating*sex interaction p-value			0.681

List of abbreviations: CI = confidence interval Adjusted model 1: sex, ethnicity, education, marital status, social class, total CIS-R score, smoking, and alcohol consumption Adjusted model 2 = model 1 + number of metabolic syndrome constituent components at baseline Adjusted model 3 = model 2 + BMI at baseline

a The negative binomial regression coefficient is to be interpreted as the difference in the logs of expected counts of metabolic syndrome constituent components at phase 2 in those with binge eating at baseline compared to those without binge eating at baseline.

**Table 3:** Univariable and multivariable logistic regression models of the association between binge eating at baseline and individual constituent component of the metabolic syndrome at follow up. Sample based on participants with complete data. (n=13,388)

	Hypertension	Hypertriglyceridemia	High fasting blood glucose	Low HDL cholesterol	High waist circumference
	**n (%) with outcome**	**n (%) with outcome**	**n (%) with outcome**	**n (%) with outcome**	**n (%) with outcome**
*Binge eating absent*	4,902 (43.2%)	3,091 (27.3%)	2,175 (19.2%)	2,776 (24.5%)	8,438 (74.3%)
*Binge eating present*	1,001 (48.9%)	716 (35.0%)	500 (24.4%)	654 (31.9%)	1,812 (88.5%)
	**Odds ratio (95%CI)**	**Odds ratio (95%CI)**	**Odds ratio (95%CI)**	**Odds ratio (95%CI)**	**Odds ratio (95%CI)**
Crude model	1.26 (1.14 to 1.38), p<0.0001	1.43 (1.30 to 1.58), p<0.0001	1.36 (1.22 to 1.52), p<0.0001	1.44 (1.31 to 1.60), p<0.0001	2.65 (2.30 to 3.06), p<0.0001
Adjusted model 1	1.41 (1.27 to 1.56), p<0.0001	1.50 (1.35 to 1.67), P<0.0001	1.48 (1.32 to 1.67), p<0.0001	1.32 (1.19 to 1.47), p<0.0001	2.57 (2.23 to 2.97), p<0.0001
Adjusted model 2	1.41 (1.23 to 1.62), p<0.0001	1.33 (1.17 to 1.50), P<0.0001	1.27 (1.09 to 1.50), p=0.003	1.22 (1.08 to 1.38), p=0.002	1.39 (1.16 to 1.68), p=0.0004
Adjusted model 3	1.14 (0.99 to 1.31), p=0.067	1.21 (1.07 to 1.37), p=0.003	1.00 (0.85 to 1.18), p=0.989	1.06 (0.93 to 1.21), p=0.364	0.92 (0.75 to 1.13), p=0.449
Binge eating*sex interaction p-value	0.577	0.020	0.229	0.773	0.745
Males		1.39 (1.15 to 1.68)			
Females		1.09 (0.92 to 1.29)			

List of abbreviations: CI = confidence interval Adjusted model 1: sex, ethnicity, education, marital status, social class, total CIS-R score, smoking, and alcohol consumption Adjusted model 2 = model 1 + outcome value at baseline Adjusted model 3 = model 2 + BMI at baseline

## Data Availability

For information on data availability please estatisticaelsa@ufrgs.br

## Significant outcomes

- Adults reporting weekly episodes of binge eating had greater odds of having metabolic syndrome at follow up, although this association was explained by higher BMI at baseline.
- Adults reporting weekly episodes of binge eating had higher odds of having hypertriglyceridemia and hypertension at follow up independently of baseline BMI.

## Limitations

- The question on binge eating behaviours could not differentiate between subjective and objective binge eating.
- The association between binge eating and hypertension was weak possibly due to low statistical power.
- We could not explore mechanisms of this association, as we only had two time points of data available.
