# Supplementary material for: Longitudinal association between binge eating and metabolic syndrome in adults: findings from the ELSA-Brasil cohort
Source: Acta Psychiatr Scand. Author manuscript; Available in PMC 2022 Aug 5. (PMC7613243; doi:10.1111/acps.13356)
Supplement: Supplemental material [file EMS151981-supplement-Supplemental_material.pdf]

Longitudinal association between binge eating and metabolic syndrome in adults: findings from the ELSA-Brasil cohort.

Francesca Solmi PhD[1], Arlinda B. Moreno PhD[2*], Glyn Lewis PhD[1], Maria Angélica Nunes PhD[3], Maria de Jesus Mendes da Fonseca PhD[2], Rosane Harter Griep PhD[4]*

[1] *Division of Psychiatry, University College London, London, UK*

[2] *Department of Epidemiology and Quantitative Methods in Health, National School of Public Health, Oswaldo Cruz Foundation, Rio de Janeiro, Brazil*

[3] *Laboratory of Health and Environment Education, Oswaldo Cruz Institute, Oswaldo Cruz Foundation, Rio de Janeiro, Brazil*

[4] *Postgraduate Program in Epidemiology, School of Medicine, Federal University of Rio Grande do Sul, Porto Alegre, RS, Brazil*

Corresponding author:

Francesca Solmi, PhD

UCL Division of Psychiatry,

Maple House, 6th Floor, Wing A

149 Tottenham Court Road,

W1T 7NF, London, UK

Email: Francesca.solmi@ucl.ac.uk, elsa@fiocruz.br

Phone: +44(0)2076799643

Contents.. 2

eFigure 1: Direct Acyclic Graph showing our causal assumption.. 3

eFigure 2: Flowchart of study participation 4

eTable 1: Predictor of missingness at follow up among those with complete exposure (n=15,074) 5

eTable 2: Univariable and multivariable logistic regression models of the association between binge eating at baseline and Metabolic syndrome at follow up. Sample based on those with complete exposure and imputed confounders and outcomes. Sample of participants with complete exposure and imputed outcome and confounders who were alive at follow up (n=14,828)... 6

eTable 3: Univariable and multivariable logistic regression models of the association between binge eating at baseline and individual symptoms of Metabolic syndrome at phase two. Sample of participants with complete exposure and imputed outcome and confounders who were alive at follow up (n=14,828) 7

eFigure 1: Direct Acyclic Graph showing our causal assumption

eFigure 1a

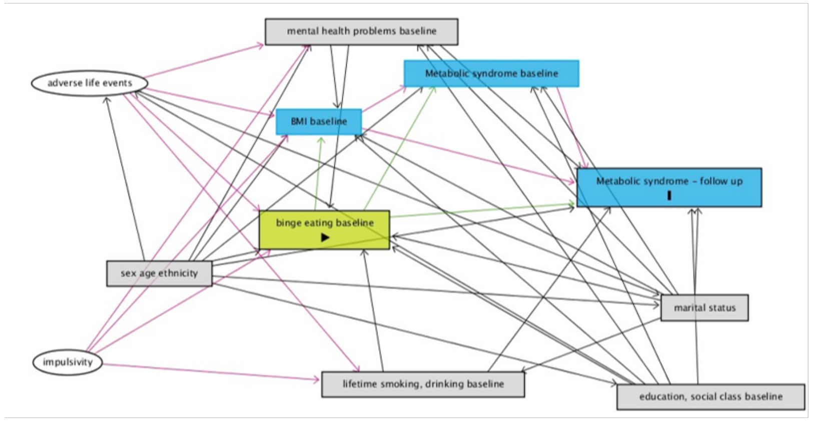

eFigure 1b

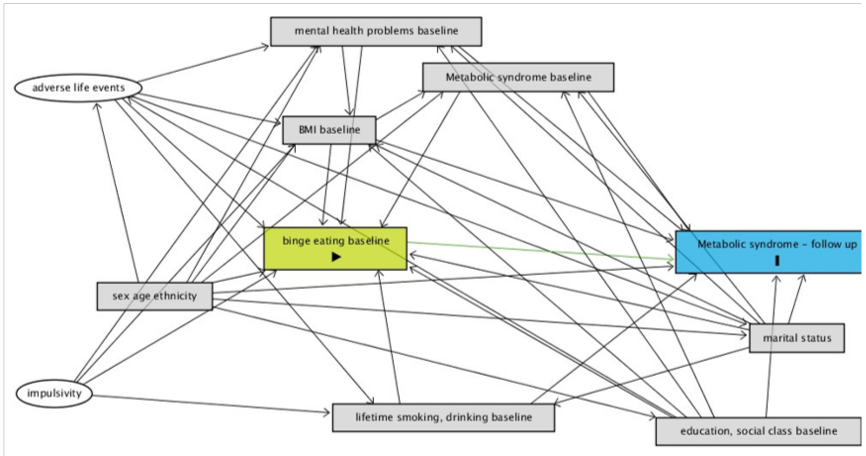

Legend: In these DAGs the green rectangle represents the exposure (i.e. binge eating at baseline) and the blue rectangle with black borders is the outcome (i.e. metabolic syndrome at follow up). In the figure, grey and black lines represent biasing paths that have been controlled for by adjustment for observed confounders; pink arrows represent biasing paths that are not controlled for (in eFigure 1a for example because impulsivity and stressful events are not controlled for as we did not have that information). Green arrows represent causal paths.

All other variables in rectangles and circles represent potential confounders, mediators, and the associations between them, which we explored. Based on these assumptions, in order to estimate the direct effect of the exposure and the outcome, it is sufficient to adjust for participants' sex, age, ethnicity, highest education, social class, mental health problems at baseline, smoking and drinking behaviours, and marital status. We had two alternative hypotheses regarding the role of BMI and metabolic syndrome at baseline: either they could be mediators of the association (Figure 1a) or confounders (Figure 1b). To estimate the direct effect, we also needed to account for these two factors. Given the cross-sectional nature of their measurement at baseline, we included as confounders.

Variables in grey rectangles are those we will be able to control (although in figure 1a they have been left in blue for clarity regarding their hypothesised role as mediators) and those in white circles those that are unobserved, but also did not need to be adjusted for.

eFigure 2: Flowchart of study participation

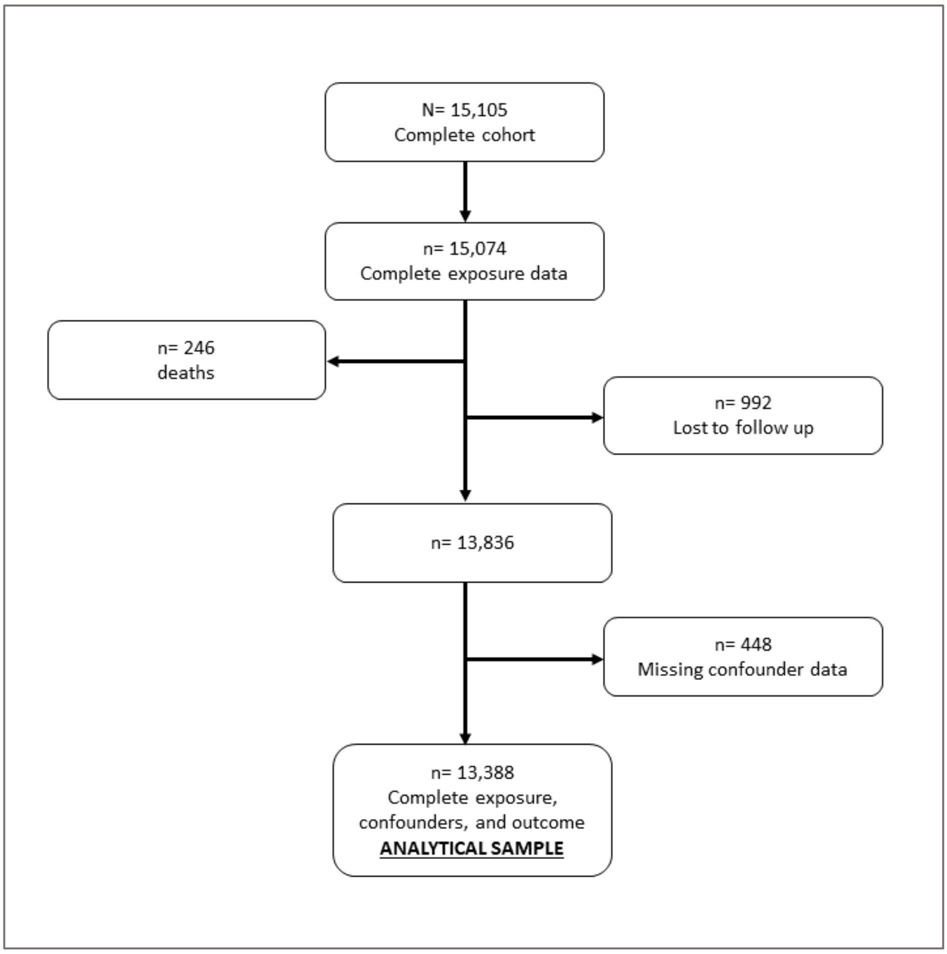

eTable 1: Predictor of missingness at follow up among those with complete exposure (n=15,074)

	Reason for non-participation at follow up	
	**Lost to follow up OR (95%CI)**	**Death (OR 95%CI)**
	992 (6.6%)	246 (1.6%)
**Binge eating**		
Absent	Reference	Reference
Present	1.07 (0.90 to 1.28)	0.91 (0.64 to 1.32)
**Sex**		
Male	Reference	Reference
Female	0.93 (0.82 to 1.06)	0.62 (0.48 to 0.80)
**Ethnicity**		
Black	Reference	Reference
Pardo	1.17 (0.96 to 1.43)	0.74 (0.51 to 1.06)
White	0.95 (0.78 to 1.15)	0.64 (0.46 to 0.90)
Asian or indigenous	0.87 (0.58 to 1.30)	0.76 (0.37 to 1.56)
**Highest education**		
No schooling	Reference	Reference
Elementary school	0.84 (0.62 to 1.13)	0.49 (0.31 to 0.78)
Secondary school	0.54 (0.43 to 0.69)	0.23 (0.16 to 0.34)
University degree	0.44 (0.35 to 0.55)	0.20 (0.14 to 0.29)
**Marital status**		
Married	Reference	Reference
Partner	1.23 (1.04 to 1.47)	0.84 (0.57 to 1.24)
Separated/divorced	1.12 (0.94 to 1.33)	1.12 (0.80 to 1.57)
Single	1.09 (0.87 to 1.37)	1.01 (0.65 to 1.57)
Widowed	1.63 (1.22 to 2.16)	2.42 (1.53 to 3.81)
**Social Class**		
Manual-routine	Reference	Reference
Manual non-routine	1.13 (0.68 to 1.87)	2.37 (1.26 to 4.47)
Non-manual routine	0.73 (0.60 to 0.87)	0.59 (0.42 to 0.83)
Non manual non routine	0.71 (0.60 to 0.84)	0.47 (0.34 to 0.64)
**Smoker**		
Never smoker	Reference	Reference
Past smoker	1.22 (1.06 to 1.41)	1.50 (1.12 to 2.03)
Current smoker	1.44 (1.50 to 2.08)	3.11 (2.28 to 4.24)
**Alcohol use**		
Never drank	Reference	Reference
Past drinker	0.90 (0.73 to 1.12)	1.68 (1.04 to 2.72)
Current drinker	0.67 (0.55 to 0.81)	1.09 (0.69 to 1.71)
**Metabolic syndrome baseline**		
No	Reference	Reference
Yes	1.36 (1.18 to 1.56)	1.75 (1.35 to 2.26)
**Age**	1.03 (1.02 to 1.03)	1.08 (1.06 to 1.09)
**Body Mass Index**	1.03 (1.01 to 1.04)	0.99 (0.98 to 1.01)
**CIS-R-total score**	1.01 (1.00 to 1.02)	0.99 (0.98 to 1.01)

eTable 2: Univariable and multivariable logistic regression models of the association between binge eating at baseline and Metabolic syndrome at follow up. Sample based on those with complete exposure and imputed confounders and outcomes. Sample of participants with complete exposure and imputed outcome and confounders who were alive at follow up (n=14,828)

	**Odds ratio (95%CI)**
Crude model	1.59 (1.45 to 1.75), p<0.0001
Adjusted model 1	1.68 (1.53 to 1.86), p<0.0001
Adjusted model 2	1.14 (1.02 to 1.27), p=0.02
Adjusted model 3	1.09 (0.96 to 1.24), p=0.17
Binge eating*sex interaction p=value	0.738

Adjusted model 1: sex, ethnicity, education, marital status, social class, total CIS-R score, smoking, and alcohol consumption
Adjusted model 2 = model 1 + Metabolic syndrome at baseline
Adjusted model 3 = model 2 + BMI at baseline

eTable 3: Univariable and multivariable logistic regression models of the association between binge eating at baseline and individual symptoms of Metabolic syndrome at phase two. Sample of participants with complete exposure and imputed outcome and confounders who were alive at follow up (n=14,828)

	**Hypertension**	**Hypertriglyceridemia**	**High fasting blood glucose**	**Low HDL cholesterol**	**High waist circumference**
	**OR (95%CI)**	**OR (95%CI)**	**OR (95%CI)**	**OR (95%CI)**	**OR (95%CI)**
Crude model	1.26 (1.15 to 1.37), p<0.0001	1.43 (1.30 to 1.58), p<0.0001	1.37 (1.22 to 1.52), p<0.0001	1.45 (1.31 to 1.60), p<0.0001	2.59 (2.26 to 2.97), p<0.0001
Adjusted model 1	1.43 (1.29 to 1.57), p<0.0001	1.51 (1.36 to 1.67), P<0.0001	1.50 (1.34 to 1.69), p<0.0001	1.33 (1.20 to 1.47), p<0.0001	2.53 (2.21 to 2.92), p<0.0001
Adjusted model 2	1.43 (1.25 to 1.63), p<0.0001	1.33 (1.17 to 1.50), P<0.0001	1.27 (1.07 to 1.47), p=0.004	1.22 (1.08 to 1.38), p=0.001	1.37 (1.14 to 1.64), p=0.001
Adjusted model 3	1.15 (1.00 to 1.32), p=0.055	1.21 (1.07 to 1.37), P=0.003	0.98 (0.84 to 1.16), p=0.847	1.06 (0.93 to 1.21), p=0.365	0.91 (0.74 to 1.11), p=0.364
Binge eating*sex interaction p=value	p=0.542	p=0.053	p=0.613	p=0.852	p=0.721
Males		1.35 (1.12 to 1.63)			
Females		1.11 (0.94 to 1.31)			

Adjusted model 1: sex, ethnicity, education, marital status, social class, total CIS-R score, smoking, and alcohol consumption
Adjusted model 2 = model 1 + outcome value at baseline
Adjusted model 3 = model 2 + BMI at baseline
